# Kerosene Oil Poisoning among Children in Rural Sri Lanka

**DOI:** 10.1155/2017/8798610

**Published:** 2017-11-16

**Authors:** M. B. Kavinda Chandimal Dayasiri, Shaluka F. Jayamanne, Chamilka Y. Jayasinghe

**Affiliations:** ^1^Department of Paediatrics, Oxford University Hospitals NHS Foundation Trust, Oxford, UK; ^2^Faculty of Medicine, University of Kelaniya, Kelaniya, Sri Lanka

## Abstract

**Introduction:**

Kerosene oil poisoning is one of common presentations to emergency departments among children in rural territories of developing countries. This study aimed to describe clinical manifestations, reasons for delayed presentations, harmful first aid practices, complications, and risk factors related to kerosene oil poisoning among children in rural Sri Lanka.

**Methods:**

This multicenter study was conducted in North-Central province of Sri Lanka involving all in-patient children with acute kerosene oil poisoning. Data were collected over seven years from thirty-six hospitals in the province. Data collection was done by pretested, multistructured questionnaires and a qualitative study.

**Results:**

Male children accounted for 189 (60.4%) while 283 (93%) children were below five years. The majority of parents belonged to farming community. Most children ingested kerosene oil in home kitchen. Mortality rate was 0.3%. Lack of transport facilities and financial resources were common reasons for delayed management. Hospital transfer rate was 65.5%. Thirty percent of caregivers practiced harmful first aid measures. Commonest complication was chemical pneumonitis. Strongest risk factors for kerosene oil poisoning were unsafe storage, inadequate supervision, and inadequate house space.

**Conclusions:**

Effect of safe storage and community education in reducing the burden of kerosene oil poisoning should be evaluated. Since many risk factors interact to bring about the event of poisoning in a child, holistic approaches to community education in rural settings are recommended.

## 1. Introduction

Acute poisoning in children adversely impacts on global child health and the patterns and morbidity of poisoning vary across different geographic regions due to variable cultural, social, economic, and geographic factors. Globally more than one million children die following injuries every year [1] and poisoning is identified as the fourth leading cause of injury related mortality in children [2]. Children younger than five years have the highest risk for acute poisoning [3] while the majority of them belong to either lower or middle income countries [2].

Kerosene oil poisoning in children is a preventable cause of significant mortality and morbidity. Kerosene oil is a liquid hydrocarbon and its toxicity depends on constituent naphthenic and aromatic hydrocarbons and low viscosity. Primarily it causes pulmonary complications including chemical pneumonitis though the central nervous system and ventilator drive can also be adversely affected by its effect on myelin. Children can be intoxicated by ingestion, inhalation, or contact of kerosene oil with skin. Though the developed countries have largely eliminated accidental kerosene ingestions [4], kerosene oil remains to be the commonest poisoning substance among children in many South Asian (India [5], Pakistan [6], Nepal [7], and Bangladesh [8]) and African (Nigeria [9], Kenya [10], and Zimbabwe [11]) countries. Low socioeconomic status, unsafe storage, and large family size are previously reported risk factors for kerosene oil poisoning among children [6] though these risk factors may change across different sociogeographic locations worldwide.

Evidence on kerosene oil poisoning among children in rural Sri Lanka is sparse. The prospective study by Lucas GN [12] evaluated children with kerosene oil poisoning from a predominant urban population two decades ago. Currently, the advancement of quality of life and increased use of electricity and liquefied petroleum gas have decreased kerosene oil poisoning events in more urban communities of Sri Lanka. To date there are no studies on children with kerosene oil poisoning in rural Sri Lanka where kerosene oil is increasingly used as a source of low-cost cooking and lighting fuel and a heating and cleaning agent. Moreover, there is no evidence from either prospective or retrospective studies on patterns and risk factors related to kerosene oil poisoning among children in rural Sri Lanka. The objective of the current study was to comprehensively evaluate the patient profiles, circumstances of poisoning, symptom profile and complications, reasons for delayed management, harmful first aid measures, and risk factors for kerosene oil poisoning in children of rural Sri Lanka.

## 2. Methods

### 2.1. Study Setting

This multicenter study was hospital based and conducted in the North-Central province of Sri Lanka. A predominantly rural population resides in this province while the majority of them belong to farming community. The province accommodates a population of 1,259,567 (Anuradhapura district: 856,232 and Polonnaruwa district: 403,335) while 29.2% are less than 14 years [13]. Kerosene oil is widely used both as cooking fuel and lighting fuel. The study was conducted in the two major hospitals of the province which function as referral centers for the entire province and they were Anuradhapura teaching hospital and Polonnaruwa District General hospital. In addition, data were collected from thirty-four other regional hospitals which function under RDHS (Regional Director of Health Services) of the North-Central province and these hospitals receive children from the most remote territories in the province.

### 2.2. Study Design

Data collections in the current study were done by both structured interviews and focused group discussions. Data collected in the retrospective study were observational. The time period covered by the study was seven years (2007 February–2014 January). The study was conducted in four major arms: (1) a two-year prospective study (2012 February–2014 January) at Anuradhapura teaching hospital (TH), (2) a two-year prospective study (2012 February–2014 January) at Polonnaruwa District General hospital (DGH), (3) one-year prospective study at thirty-four regional hospitals within RDHS of NCP (2013 January–2014 January), and (4) a five-year retrospective study at Anuradhapura teaching hospital (2007 February–2012 January).

### 2.3. Participants

This study recruited all in-patient children who presented with either acute unintentional or intentional poisoning of kerosene oil. Children were recruited to the observational study after their poisoning events were confirmed by care givers following the initial evaluation at the hospital emergency department and subsequently at general paediatric wards. All children who were between 9 months to 12 years of age were recruited to the study. Poisoning with other household chemicals, plants, pesticides, and medicines, food poisoning, snake envenomation, allergic reactions, and adverse drug reactions which can be considered in the purview of toxicology were omitted in the study. Children with doubtful poisoning with kerosene oil were also excluded from the study.

### 2.4. Data Collection

Data were collected from the caregivers of children who met inclusion criteria. Mothers were interviewed in most encounters and fathers or other caregivers (grandparents and other related caregivers) were interviewed only when mothers were not available to participate in the study. Major part of the data collection was conducted at Anuradhapura teaching hospital and data collection from all caregivers in the prospective study in that setting was done by the principal investigator himself to minimize interviewer bias. Interviews with the caregivers in the prospective study were conducted on the same day of admission to minimize possible recall bias. Data were collected using a pretested multistructured questionnaire which comprised questions to identify demographic data, type, and circumstances of poisoning, poison related factors, location of poisoning, harmful first aid measures, clinical management, and reasons for delayed management. The complications and outcomes following acute poisoning were recorded from Bed Head Tickets (BHT) at the time of discharge of the child from the study setting. The questionnaire was pretested by administration of the questionnaire to twenty caregivers in the same study setting over four-month period prior to commencement of the study and expert review. Extensive local and international literature survey was done prior to drafting of the questionnaire. Clinical research associates carried out data collection at Polonnaruwa DGH and local hospitals under RDHS. All clinical research associates were trained by the principal investigator to administer questionnaires to minimize interviewer bias. Piloting was carried out in all study settings for four months prior to commencement of the study and all data collections were done under direct supervision of the investigators of the study. Retrospective study was conducted based on Bed head ticket data and only limited demography and poison factor related data which could be considered reliable and auditable by discharge registers were collected. Data in the retrospective series were collected by principal investigator himself to minimize record retrieval related bias.

A prospective controlled risk factor study included all children who presented with kerosene oil poisoning to Anuradhapura teaching hospital over the two-year study period (2012 February–2014 January). The risk factors were determined based on qualitative evaluation of the parents of children with kerosene oil poisoning over four months at the pretesting stage. Relevant medical literature was searched and expert review was done prior to confirming proposed risk factors. The controls were selected from the same hospital and children who presented with acute medical illnesses were recruited as controls. The acute medical illnesses considered included viral fever, acute upper respiratory tract infection, and urticaria. All other acute conditions including nonspecific symptoms without a definitive diagnosis were excluded. All children were matched for age and gender on individual patient basis. All data in the risk factors survey were collected by the principal investigator himself to minimize interviewer bias.

In order to perform an in-depth analysis of predisposing risk factors of kerosene oil poisoning and adverse first aid measures, a qualitative study was conducted by the principal investigator recruiting all children with kerosene oil poisoning and their parents at Anuradhapura teaching hospital. Data collection was done prospectively over two years via focused group discussions (FGD) and parents' narrative in the phenomenological study design. In retrospect, the qualitative data were grouped to three interrelated domains of enquiry: (1) presence of child, parent, and environment related risk factors, (2) issues related to first aid care and provision of care until child was brought to emergency care unit, and (3) possible measures to prevent further poisoning. All FGDs were carried out by the same investigator to minimize information retrieval bias and to assimilate an in depth and rich knowledge regarding varying sociocultural, economic, and parental concerns leading to accidental kerosene oil poisoning among children. Data generated through FGDs were recorded as field notes with quotations where relevant. Important parent opinions regarding all three domains were recorded.

### 2.5. Data Analysis

All quantitative data were analysed using SPSS version 19.0.

### 2.6. Data Reliability and Auditing

Data collections in all components of the current study were subjected to independent audit and close monitoring by South Asian Clinical Toxicology Research Collaboration (SACTRC) and the investigators of the study.

### 2.7. Ethical Approval

Ethical clearance for the study was issued by ethical review committees, faculty of medicine, University of Kelaniya, and Rajarata University of Sri Lanka. Written informed consent was obtained from participant children's parents/guardians in the prospective study.

## 3. Results

There were 313 incidents of kerosene poisoning reported in all arms of the study. Male children outnumbered female children in all studies and amounted to 189 (60.4%). Ninety-three percent of children (283/313) were less than five years. Only eight children were aged more than 10 years (2.6%). All poisoning events with kerosene oil were secondary to unintentional ingestion of the poison (313/313, 100%). Mortality rate was 0.3% (1 case) and the reason for mortality had been severe aspiration pneumonia following intoxication and administration of water as a first aid measure. Sixty-five percent of children (205/313) were transferred from a local hospital (under RDHS) to a tertiary care hospital following the poisoning event.  Table 1 has compared the demographic characteristics and transfer rates of children in different arms of the study.

Commonest route of poisoning was ingestion (308/313, 98.4%). Five children developed symptoms following inhalation of kerosene oil. Mean duration of hospital stay was 2.1 days (range 1–12 days).

*Comparison of Clinical Manifestations and Reasons for Delayed Presentations to Primary Care Hospital*. 196 children recruited to studies at THA, THP, and RDHS were available for the analysis. Respiratory symptoms (cough/shortness of breathing/wheezing) were the predominant symptoms following kerosene intoxication (184 children, 93.9%) and it was consistently seen in all three studies. Gastrointestinal symptoms (vomiting, nausea, and abdominal pain) occurred in six children. Two children had neurological symptoms (giddiness, drowsiness). Twelve children remained asymptomatic following kerosene oil ingestion.  Table 2 illustrates the variability in clinical manifestations in detail.

Twenty-six children (13.2%) presented to primary care hospital at least two hours after the ingestion of the poison. Commonest reason for delayed presentation was lack of transport facilities, 24 children (12.2%). Lack of transport facilities and financial resources as reasons for delayed presentation were more commonly seen among children living in the rural most territory of the province, regional director of health services (RDHS) region. Detailed analysis of reasons for delayed presentation to primary care unit is presented in Table 3.

*Detailed Evaluation of Patterns and Risk Factors of Kerosene Oil Poisoning among Children at Anuradhapura Teaching Hospital*. Eighty-three children presented to THA following kerosene oil poisoning over the two-year study period. Children belonged to 26 MOH (Medical Officer of Health) and 53 PHM (Public Health Midwife) divisions of Anuradhapura district. Mean age of children was 1.9 years (range: 12 months–11 years). Most parents had received secondary education, 59 fathers (71.1%) and 65 mothers (78.3%). The majority of fathers were engaged in farming (22, 26.5%), manual labor (17, 20.4%), and small scale business (8, 9.6%). Most mothers were housewives (59, 71.1%). Most of the poisoning events occurred in home kitchen (70, 84.3%) followed by sleeping area (6, 7.2%) and home garden (5, 6%).

Harmful first aid measures were practiced in 25 children (30.1%). The commonest measure was forceful ingestion of coconut milk (18, 21.7%) and it was followed by forceful ingestion of milk (3, 3.6%) and water ingestion (2, 2.4%). Among 25 children, care givers of only five children (20%) were aware of the increased risk of aspiration following these harmful first aid measures. Emesis was not induced appropriately at the primary care unit in all cases.

The majority of children had onset of symptoms within one hour from the time of poisoning event (79, 95.2%). Though most children (65, 78.3%) were brought to primary care unit within 45 minutes from the poisoning event, fifteen children (18%) presented at least one hour after the poison was ingested (range: 1 to 6 hours). Ten cases of aspiration pneumonia/chemical pneumonitis (12%) were reported following kerosene oil ingestion. There were no reported central nervous system complications.

Risk factor evaluation showed six proposed risk factors which were associated with significantly elevated risk (*p* < 0.05) of kerosene oil poisoning among children. They were the unsafe storage of household poisons, inadequate supervision of the child, inadequate house space, subjective economic problems in the family, lack of education in mother (<primary education), and parents' subjective feeling of lack of family support to look after children. Five proposed risk factors did not reveal a significant association with kerosene oil poisoning (working mother, children with deprived schooling, young mother, farming parents, and past history of poisoning).  Table 4 has compared the presence of proposed risk factors in the two groups.

## 4. Qualitative Study

Eighty-three care givers along with their children were recruited to focused group discussions (FGD) over the two-year study period. All discussions were carried out until a thematic saturation was achieved in information retrieval and the discussion time ranged from 5 minutes to 13 minutes. The culture in rural villages in Sri Lanka has predisposed to various scientifically unproven first aid practices that a child receives following an acute poisoning event. A parent (case 19) said “it was my mother who advised me to give coconut milk following the child developed breathing difficulty after ingesting kerosene. She believed that it may reduce the amount of poison.” There were seven incidents where parents practiced potentially harmful emesis induction measures on children as first aids and all children developed aspiration pneumonia.

It was revealed during the FGD that multiple risk factors can interact to bring about the poisoning event. A two-year old girl was brought to hospital (case 27) following accidental ingestion of kerosene oil. The care givers were from a poor social background and both parents had not received secondary education. They lived in a rented room and cooking stove was in one of the corners of the room with kerosene oil bottles being kept just on the floor. Parents had never thought of the possibility of poisoning and they had not bothered to supervise the child. It was evident, given the multitudinous issues, that mere provision of advice on safe storage would not eliminate the risk of poisoning and the risk factors need to be dealt with in a holistic way.

Inappropriate storage also can lead to accidental poisoning. Many families in rural Sri Lanka use kerosene oil for lighting lamps and cooking and to chase away venomous snakes. The researchers had twenty-three experiences with children who accidentally ingested kerosene oil which were stored in beverage and juice bottles. It was common parental opinion that majority of children who ingested kerosene oil did so in their own home kitchen area. Therefore identification of this issue and subsequent safe storage would dramatically bring down most of the poisoning events and it would be a much cost effective, feasible, and practically sound intervention.

## 5. Discussion

Kerosene oil poisoning is the commonest type of acute poisoning among children in developing countries accounting for more than 60% of poisoning events [10]. Though mortality is rare, kerosene oil poisoning leads to significant morbidity following its adverse effects on respiratory, gastrointestinal, and central nervous systems [14]. Kerosene oil damages type II pneumocytes compromising surfactant production and function. Hydrocarbon aspiration leads to intra-alveolar haemorrhage, inflammation, and necrosis. Children in current study had predominant respiratory tract related clinical manifestations and it was similarly seen in other studies [15].

Kerosene oil is the leading cause of acute poisoning among children in Sri Lanka [12]. Management of poisoning related morbidity in these patients adds significantly to country healthcare expenditure. The average cost of treating an adult patient following poisoning in a rural district of Sri Lanka was US$ 31.83 with ward staff input and medications having highest expenditure [16]. The same study revealed that total expenditure on treating self-poisoned patients in Anuradhapura teaching hospital amounted to US$ 76,599 in 2006. Current study revealed that a child receives treatment as in-patient for an average 2.1 days with 12.1% of children needing treatment for aspiration/chemical pneumonitis. Data on cost of treating children with kerosene oil poisoning in Sri Lanka is not reported though the economic loss is likely to be high with healthcare costs and loss of employment days in parents.

Another important observation in the current study was the higher transfer rate of children. The study identified that 65.5% of children were transferred from primary healthcare hospital to tertiary hospital for further management. Previously published adult studies in the same region of Sri Lanka observed a transfer rate of 50% [17]. Unintentional kerosene oil poisonings in the paediatric age group in sharp contrast to adult poisonings are associated with much favorable outcomes and complications are rarer. Transferring of patients also significantly adds to healthcare expenditure and the average patient cost per transfer was US$ 14.03 in one study eight years before in the same region [16]. These figures highlight the value of increased awareness among health care workers in rural territories regarding the mostly benign nature and outcome of kerosene poisoning in the paediatric age group. Effective triage and limitation of transfers only to needy children would likely cut down healthcare expenditure, duration of hospital stay, and effect on families of children with kerosene oil poisoning.

Patterns of poisoning and subsequent outcome are always related to the underlying sociocultural circumstances. This study revealed that 30.1% of children were offered potentially harmful first aid measures before arriving to hospital. Only 20% of caregivers were aware of the detrimental effects of their practices. Therefore providing knowledge to at risk communities regarding such issues is helpful in bringing down childhood poisoning related morbidity and mortality. Two decades ago, Sri Lankan studies reported that 67% of children were offered harmful first aid measures following kerosene oil ingestion [18]. The decrease observed over time could be secondary to increased parental education, community awareness, and improvement of social standards. Authors suggest further reduction of incidence through well executed community education interventions.

Current study did not observe intentional ingestions of kerosene oil in contrast with previous Sri Lankan studies [18]. Mortality was also less as compared with previous studies [18]. Delayed presentation to primary care hospital following the poisoning event has a potentially strong negative impact on effective management and patient outcomes [19]. In the current study, the commonest reasons for delayed presentation were lack of transport facilities and financial resources. Kerosene oil is used mostly by caregivers with poor socioeconomic backgrounds. Electricity and established transport facilities are not available in some rural territories. The duration of hospital stay and severity of complications have been shown to have a direct correlation with lag time in reaching the hospital following the poisoning event [20]. These facts reveal the value of community awareness in seeking early primary care in improving patient outcomes.

Risk factor evaluation in current study revealed unsafe storage, inadequate supervision, inadequate house space, economic problems, lack of education in mother, and lack of family support as significant risk factors leading to the poisoning event by kerosene oil ingestion. Age and gender were matched by individual patient basis. Previous South Asian studies reported male gender, young age (<2 years), large family size, poor socioeconomic status, and unsafe storage as risk factors for kerosene oil poisoning [21]. The current study observed proportionally higher number of male and < two-year-old children as compared with other age groups and female gender. This observation is similar in other studies [22]. Poor socioeconomic status and inadequate house space were reported as risk factors for kerosene oil poisoning in studies from Africa [23] and Middle East [24]. It was understood in the qualitative study that many of these risk factors interact to bring about the event of poisoning rather than the effect of a single risk factor.

Kerosene oil poisoning mostly occurs in resource limited settings where it is frequently used. Children are easily attracted by colourful packaging [25]. Twenty-seven percent of caregivers in the current study stored them in beverage/juice bottles. Unsafe storage was the most significant risk factor for poisoning events. Studies have suggested addition of blue dye [26], child-proofed containers [27], and community education [28] as measures of prevention of child exposure to kerosene. Since the burden of kerosene oil poisoning is largely preventable evaluation of effectiveness of these interventions in this population by public health studies is a need of preventive healthcare. As most poisoning events occurred within home premises in the current study, a holistic approach which targets home environment together with empowerment of parents would help in managing the burden of kerosene oil poisoning in this community.

One limitation was that the study was a hospital based study. Evaluation of risk factors in current study may not represent precisely accurate picture of the community for several reasons. It is likely that the study has not addressed the poisoning events which were not brought for medical attention during the period under study. Though most children with acute poisoning are transferred from local hospitals to the teaching hospital for further management, a fraction of acute poisoning cases is likely to be not transferred, thus being not taken into account in the current study.

## 6. Conclusion

Kerosene oil poisoning is a common type of acute poisoning among children in rural Sri Lanka. Though it is associated with very low mortality, the poisoning events are associated with morbidity and burden to healthcare expenditure of the country. Harmful effects of traditional first aid practices prevalent among rural populations are detrimental to health of the child and the majority is unaware of these adverse effects. Lack of community awareness in seeking early primary care is one of the main barriers for timely management. Since unsafe storage, inadequate supervision, and inadequate house space are strongest risk factors for kerosene oil poisoning, the effect of safe storage and improved community education in reducing the burden of kerosene oil poisoning should be evaluated.

The study also identified the potential value of awareness among health care workers regarding the outcomes of kerosene oil poisoning in children in view of reducing transfer rates among hospitals.

## Figures and Tables

**Table 1:** Demographic characteristics and transfer rates of children with kerosene oil poisoning.

Variable	Retrospective study (*n* = 117)	THA study (*n* = 83)	Polonnaruwa study (*n* = 57)	Peripheral study (*n* = 56)	Total (*n* = 313)
(1) Male : female	73 : 44 62.4% : 37.6%	46 : 37 55.4% : 44.6%	35 : 22 61.4% : 38.6%	35 : 21 62.5% : 37.5%	189 : 124 60.4% : 39.6%
(2) <5 years : >5 years	108 : 9 92.3% : 7.7%	78 : 5 94% : 6%	54 : 3 94.7% : 5.3%	51 : 5 91.1% : 8.9%	283 : 34 93% : 7%
(3) Mortality	1 (0.9%)	—	—	—	1 (0.3%)
(4) Transfer rate	73 (62.4%)	60 (72.3%)	36 (63.2%)	36 (64.3%)	205 (65.5%)

**Table 2:** Clinical manifestations of kerosene oil poisoning among children in rural Sri Lanka.

Systemic clinical manifestations	THA	THP	RDHS	Total
(1) Respiratory symptoms	77 (92.7%)	53 (93%)	54 (96.4%)	184 (93.8%)
(2) Gastrointestinal symptoms	3 (3.6%)	2 (3.5%)	1 (1.8%)	6 (3.1%)
(3) Neurological symptoms	1 (1.2%)	1 (1.7%)	—	2 (1.0%)
(4) Cardiovascular symptoms	—	1 (1.7%)	—	1 (0.5%)

**Table 3:** Reasons for delayed presentation to primary care unit among children following kerosene oil poisoning in rural Sri Lanka.

Reasons for delayed presentation	THA	THP	RDHS	Total
(1) Lack of transport facilities in emergencies	7 (8.4%)	5 (8.7%)	12 (21.4%)	24 (12.2%)
(2) Lack of concern regarding urgency of the situation	9 (10.8%)	7 (12.2%)	6 (10.7%)	22 (11.2%)
(3) Lack of knowledge regarding possible complications	7 (8.4%)	6 (10.5%)	4 (7.1%)	17 (8.6%)
(4) Lack of financial resources	4 (4.8%)	5 (8.7%)	7 (12.5%)	16 (8.1%)
(5) Child had not told about incident until symptoms occur	—	—	1 (1.7%)	1 (0.5%)
(6) Delayed attention by the medical team	1 (1.7%)	—	—	1 (0.5%)

**Table 4:** Analysis of proposed risk factors in case-control study.

Proposed risk factor	Cases	Controls	Odds ratio	95% CI (OR)		*p* value
				Low	High	
(1) Unsafe storage of household poisons	76	12	3.26	2.10	4.86	<0.001
(2) Inadequate supervision of the child	74	21	3.15	1.56	4.97	<0.001
(3) Inadequate house space	41	9	4.02	2.96	5.22	<0.001
(4) Lack of family support	38	12	2.14	1.88	3.36	<0.001
(5) Subjective economic problems in the family	49	21	0.57	0.39	0.79	<0.001
(6) Lack of schooling/education in mother	17	3	3.32	1.28	7.46	<0.001
(7) Young mother (<19 years)	9	7	1.38	0.51	2.1	>0.05
(8) Children with deprived schooling	2	1	1.22	0.87	1.45	>0.05
(9) Past history of poisoning	4	5	0.96	0.64	1.42	>0.05
(10) Employed mother	24	26	0.93	0.04	1.66	>0.05
(11) Farming parents	22	28	0.76	0.14	1.21	>0.05

## Data Access

The datasets generated and/or analysed during the current study are not publicly available due to confidentiality agreement with participants but are available from the corresponding author on reasonable request.
